# The Application of Intra-Articulr Injections for Management of the Consequences of Disc Displacement without Reduction

**DOI:** 10.3390/ijerph17134726

**Published:** 2020-07-01

**Authors:** Malgorzata Pihut, Andrzej Gala

**Affiliations:** Prosthodontics Department, Consulting Room of Temporomandibular Disorders, Jagiellonian University, Medical College, 4 Montelupich Str., 31-155 Krakow, Poland; malgorzata.pihut@uj.edu.pl

**Keywords:** temporomandibular disorders, hyaluronic acid, plate rich plasma, intraarticular injection

## Abstract

The aim of the study was to make a comparative studies on the effectiveness of platelet rich plasma (PRP) and hyaluronic acid (HA) in intra-articular injections to the temporomandibular joints—in double blind studies application—based on the analysis of selected clinical parameters of functional efficiency and the mean value of joint’s pain intensity before and after management. The study enrolled a group of 100 patients, aged 21 to 43 years, of both sexes, who came for the prosthodontic treatment. All patients had II b group of disorder according to the Research Diagnostic Criteria/Temporomandibular Disorder, and were consecutively, alternately assigned to the groups, 50 patients in each. Study group PRP was treated with intra-articular injection of platelet rich plasma and study group HA had injection with hyaluronic acid. The examination was double-blind, so that the injecting physician and the patient were not informed what kind of medicinal substance they received in the joint injection. The final selected clinical parameters did not differ statistically significantly between the groups, what means that both administered substances were effective in the repair of intra-articular structures. The results of research showed that the use of PRP and HA in intraarticular joint’s injections positively affects in selected clinical parameters and decrease of the pain in temporomandibular joints in the case of disc displacement without reduction.

## 1. Introduction

Temporomandibular disorders (TMD) are an abnormality in the functioning of the masticatory muscles, temporomandibular joints involved in dynamic movements of the jaw and surrounding structures, which may be accompanied by abnormal occlusion. [1,2,3,4]. One of the most advanced form of TMD is the disc displacement without reduction. It is manifested in significant joint pain, a limited range of mouth opening (usually 20–32 mm), and a deviation of the mandible during the movement of the abduction towards the joint with a blocked disc [1,3]. Authors believe that the one of the important causes of this pathology is clenching or grinding of the teeth [2,4,5,6,7,8,9]. The reason for the occurrence of clicking may be excessive joint overload, lengthening of the capsular ligaments with simultaneous trimming of the discs [1,2,5].

The main therapeutic procedures consisted of active unblocking of the disc, due to accelerated healing of the damaged retrodiscal ligament [1]. For this purpose, joint injections of platelet-rich plasma (PRP) or hyaluronic acid (HA) into the area of the upper level of the joint were administered. Both these substances have been widely used in orthopedic and traumatic treatment with injection methods for many years [7,8,9,10,11,12,13,14].

Platelet-derived growth factors in concentrate are made of peptides that stimulate proliferation, differentiation, and migration of cells. These properties determine the usefulness of 32 growth factors in the healing process in many structures in the human body like knees, elbow pathologies, or ankles. Most growth factors with mitogenic properties are present within the platelet-rich plasma cause an increase in the repair cell counts [8,10,15,16,17]. In experimental research, Spakova at al. showed a beneficial effect of autologous PRP on inflammation of the knee [18]. Sanches [7] compared the effectiveness of PRP and HA in injections into the knee joints and showed high effectiveness in remission of the knee’s pain, despite the prevalence of PRP. On the other hand, HA is an organic chemical, a polysaccharide from the glycosaminoglycan group. It is a biopolymer with high healing properties, originally it was obtained from rooster combs, whereas nowadays it is produced using laboratory methods [7]. HA is a polysaccharide that occurs in the human body and all living organisms. We can find it, for example, in the extracellular matrix of connective tissues, cartilage, synovial fluid, vitreous body and tear film, kidneys or vocal cords, among others. It is also a component of body fluids and a building block of blood vessel walls [8,15]. When administered to the joints, it additionally increases the joint smear, and enables a much better slip of the joint heads on the disc [15,17,18]. HA is a substance with high healing properties and often used in orthopedic treatment [8,10].

The aim of the study was to make a comparative studies on the effectiveness of PRP and HA in intra-articular injections to the temporomandibular joints—in double blind studies application—based on the analysis of selected clinical parameters of functional efficiency and the mean value of joint’s pain intensity before and after management.

## 2. Material and Methods

### 2.1. Material

The study included a group of 100 patients, aged 21 to 43 years, of both genders, who came to the prosthodontic treatment in Consulting Room of Temporomandibular Disorder at Dental Institute of Jagiellonian University in Krakow, between January 2014 to February 2017, showing signs of disc displacement without reduction and with acute pain in the area of one joints. The PRP group consisted of the patients who received an intraarticular injection of PRP to temporomandibular joints. The HA group had an injection with hyaluronic acid.

Symptoms persisted from 10 days to 2 months prior to beginning of the management. The qualification of patients to participate in the research project was based on appropriate diagnostics of TMD form (II b according to RDC-/TMD), which was one of the criteria for inclusion in the research [4]. During the first visit, contraindications to the planned method of treatment with joint injections were also excluded. All patients had II b group and were consecutively, alternately assigned to the both groups, 50 patients in each. Patients were assigned to individual groups alternately, according to the order of reporting for management.

The criteria for inclusion included: pain form-(II b) of TMD (disc displacement without reduction and limitation of the extent of mandibular lowering), symptoms persisted from 10 days to 2 months prior to beginning of the treatment, no prior treatment of arthralgia, good general health, no contraindications for joint injections and PRP, and consent to participate in the research project [10,11,12,13,14,15].

The exclusion criterion included: the repeated occurrence of disc displacement without reduction; the willingness of the patient to resign from the study; the occurrence of a general disease that makes it impossible to continue the study [10,13].

The study obtained the consent of the Bioethics Committee; KBET/125/L/ 2013 of 1 July 2013 in Prosthodontic Department of Jagiellonian University Medical College in Krakow (Clinical Trials: ID122.6120.43.2016BC NCT03057262).

The required consent for taking part in this research was obtained during the first medical visit, after detailed oral and written information, in which the patients were acquainted with the purpose of the research, its course, and expected therapeutic effect. The patients also agreed to the lack of information about a specific therapeutic substance administered to the joints during the project.

### 2.2. Methods

#### 2.2.1. Functional Examination

All patients were subjected to a basic dental examination and specialist functional examination of the masticatory organ according to research diagnostic criteria RDC/TMD procedures—axis I and axis II. The first exam was conducted before treatment and second one was carried out a week after the third injection [4,6].

Patients in both groups underwent active unblocking of the articular disc by using manual manipulation. The patients were asked to move the mandible to the contralateral side of the dislocation as far as possible. From this position mouth should be opened maximally. If this manipulation was not successful it was necessary to repeat it 2–3 times, not more in one session [1].

The examination was double-blind, so that the injecting physician and the patient were not informed what kind of medicinal substance was administered into the joints. The syringes were adequately prepared, by covering the contents, as the acid is transparent, and the plasma is yellow. In all subjects, blood was taken to obtain PRP, while in HA group, the plasma was frozen for further use after the experiment. The proper syringe content was known only by the research project coordinator, not the doctor participating in the injection.

**Figure 1:**
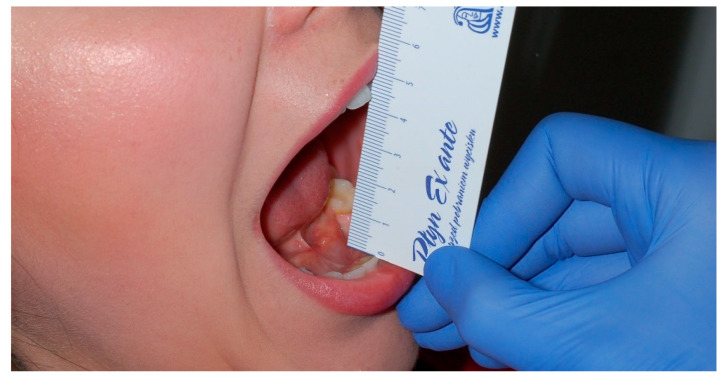
The measurement of the maximal interincisal distance.

Clinical evaluation of the effectiveness of intra-articular injections was based on the results of maximal interincisal distance, which was estimated in RDC/TMD axis I (Figure 1) [4], and subjective evaluation of the intensity of the mean value of pain experienced in the last week in the temporomandibular joints (Visual Analogue Score (VAS) from 0 to 10) RDC/TMD—Axis II.

#### 2.2.2. Intraarticular Injection

PRP injection. Peripheral blood from the ulnar vein of the patient was collected before intra-articular injections of platelet-rich plasma. Collection was done in accordance with the procedures in force using single-use, closed vacuum systems and glass tubes with sodium citrate as an anticoagulant. Mixing the collected blood thoroughly with the citrate using rotational movements preceded placing an even number of tubes in a centrifuge rotor. Centrifugation took place at 3200 rpm, and lasted for 12 min. Having separated the erythrocytes’ mass and the platelet-poor and platelet-rich plasma layered just over the erythrocytes. The platelet-rich plasma was cautiously aspirated into a separate syringe. Thus, prepared concentrate was ready for injection into the area of temporomandibular joint [18,19,20,21,22].

**Figure 2:**
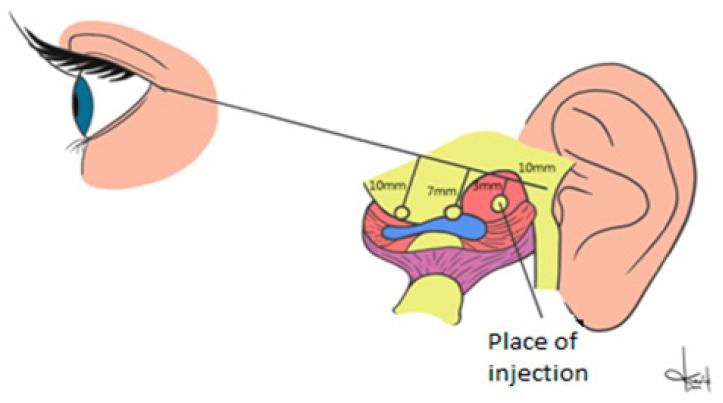
Schema of the placement of the injection point within temporomandibular joint.

Arthroscopic surgical procedures were used to determine injection spot within the temporomandibular joints for all patients in both groups (Figure 2).

**Figure 3:**
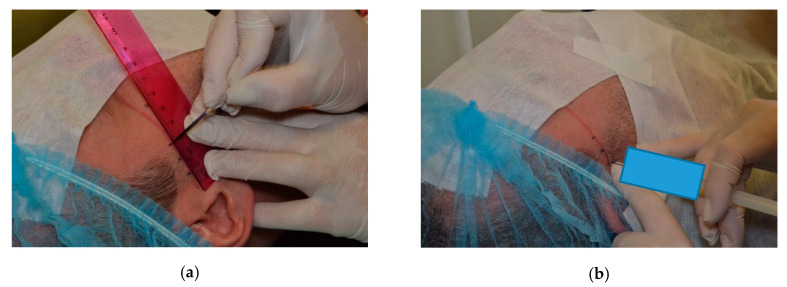
The assignation of the point of intra articular injection (**a**) the lines marked every 10 mm, (**b**) the injection was made at the end of 3 mm long line, started from the first marked line.

A disinfectant was used to wash the skin and to decontaminate the field of the injection. Injections were made in the determined point with the mandible maximal opened. A line drawn on the skin between the earlobe and the outer eye corner was used for preparing the patients for the procedure. Starting from the earlobe 10 mm intervals were used for marking three segments. The first line had 3 mm, the second 5 mm, and the third had 7 mm. The PRP injection site was selected by the top of the first line (3 mm long). It corresponded with the upper compartment and retrodiscal zone. After injection, the skin was disinfected once again. Patients were given information that experiencing an unpleasant and transient sensation of fullness or compression in the joint regions was possible [15,18]. The same procedure was used with hyaluronic acid. Each of the patient received 0.4 mL of PRP (PRP group) and 0.4 mL of the hyaluronic acid (HA group) for both joints. The procedure was repeated three times, at 10-day intervals [7,9] Figure 3a,b. Throughout the process, all patients were always assigned to the same practitioner.

### 2.3. Statistical Analysis

For statistical analysis, the software SPSS (IBM) v.26 was used. A *p*-value of less than 0.05 was deemed as statistically significant. Significant statistical differences between SG and CG groups were determined by Mann–Whitney test. Non-parametric methods were used, where normality assumptions were violated with regard to variables’ distributions (the Shapiro—Wilk test was calculated beforehand for checking distributional adequacy).

To compare the relationship between the results obtained in consecutive clinical tests, *t*-test (parametric) and non-parametric Wilcoxon signed-rank test (for comparing two related samples) were used where appriopriate. Additionally, in our research, descriptive, basic statistical analysis was calculated including variables’ mean values, standard deviations, minimal and maximal values, skewness, kurtosis, etc.

## 3. Results

The PRP group contained thirty-eight females and twelve males. The HA group had forty females and ten males. The mean age in both groups was 35.2 years maximal interincisal distance.

Table 1 presents the results of the assessment of the maximal interincisal distance, evaluated before the management and obtained during the second clinical trial, after management (joint’s injection therapy).

Considering the physiological size of the jaw opening to be from 40 mm [1], the assessment of the range of mandibular lowering motion carried out before management indicates a significant degree of limitation of this motion, since the range of the maximal interincisal distance was from 22 to 32 mm. In the majority of PRP group (92%) and HA group (84%) patients, the original size did not exceed 30 mm. The results of the clinical assessment after management, indicate that in all subjects in both groups a physiological range of jaw dilation from 40 to 46 mm was obtained.

### Results of VAS

**Figure 4:**
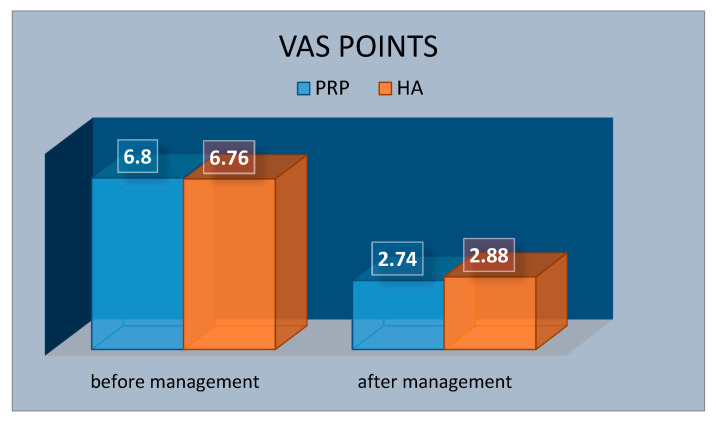
Mean value of Visual Analogue Score (VAS) points—of the temporomandibular joint’s pain before and after management in platelet rich plasma (PRP) and hyaluronic acid (HA) groups.

**Figure 5:**
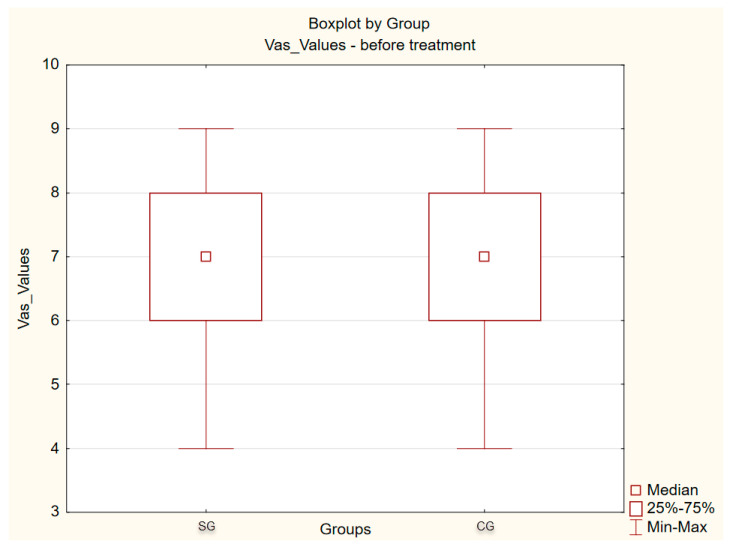
Results of VAS points before management.

**Figure 6:**
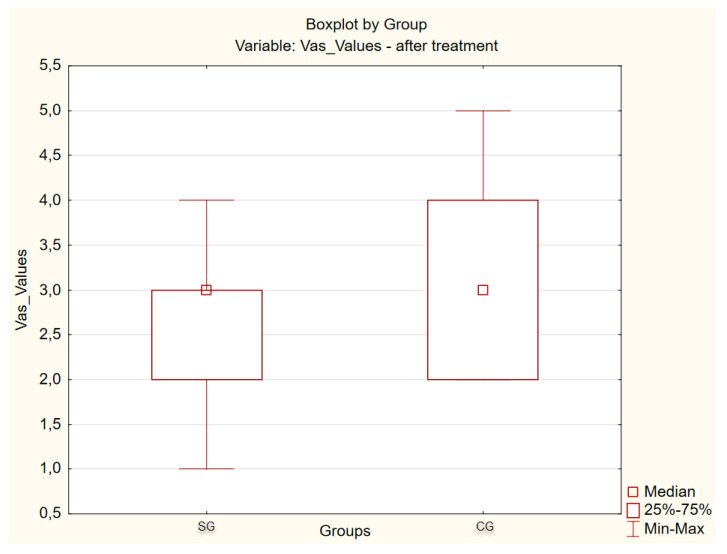
Results of VAS points after management.

Figure 4, Figure 5 and Figure 6 present the results of pain TMJ assessment using the VAS scale. The difference between mean score values obtained in PRP group (6.80) and HA group (6.76) in the VAS is not statistically significant (*p* = 0.910), which indicates homogeneity of the groups (Table 2). However, in a post-injection study, the TMJ pain score results indicate a significant reduction in joint’s pain, since, in the PRP group, the mean value of pain intensity was 2.74, and in HA group it was 2.88. These results do not differ statistically significantly between the groups, which means that both administered substances are effective in the repair of intra-articular structures. On the other hand, the results of statistical analysis comparing VAS values before and after management within both groups differ statistically significantly (*p*-value = 0.00) Table 3.

The results of statistical analysis comparing mean VAS values in exam before and after treatment within each group (PRP and HA) show a statistically significant difference (*p*-value = 0.000).

## 4. Discussion

Until today there has been no clear research results into which of the evaluated preparations is more effective in intraarticular injections. The inspiration to undertake this type of research in the past [23] were very encouraging results of own and other authors’ research concerning the use of PRP I HA in orthopedic treatment [7,9,19,20,24,25,26,27]. These authors emphasized the beneficial influence of PRP on the healing processes of bones, ligaments, joint capsule, and s-joint structures [9].

TMDs are the most common chronic orofacial pain conditions. Disc displacements without reduction are a kind of internal derangements of temporomandibular joint (TMJ). They are often responsible for pain and discomfort in the joint’s area. The literature does not provide a large spectrum of studies to perform a systematic review with subsequent meta-analysis [3,4,11].

TMD management is a complex process, due to many etiological factors that cause the disease [2,4,5,21]. Joint injections are nowadays a very valuable supplement to the basic prosthetic treatment, which is the use of occlusal splints. As confirmed by the literature, joint injections allow for the possibility of administering the drug in exactly the place that is significantly damaged in the course of TMD, which is confirmed by the literature. The preparations used for delivery injections are steroids, hyaluronic acid, and PRP, as well as the procedure of artocentesis of the TMJs. PRP and HA are substances often used in traumatology and orthopedics, due to the safety of this method, the effectiveness of the healing processes, and the improvement of the joint functioning [7,10,12,14,22,28].

The results of our study indicate a significant improvement in the range of mandibular abduction (unblocking the displaced disc) and obtaining a physiological range of mandibular abduction (i.e., above 40 mm) and a decrease in TMJ. The mean VAS scores in exam 1 and 2 within each group significantly vary statistically, which indicates the effectiveness of the procedures used. This means that three joint injections are an effective method of supporting the treatment of consequences of disc dislocation without reduction and damage to the posterior disc ligament and TMJ structures. In contrast, a comparison of the results between the groups in exam 1 and exam 2 shows that these results do not significantly differ statistically, which means that delivery injections with PRP and HA are equally effective.

Similar results were obtained by Giacomello et al. [16], who also obtained an improvement in the range of mandibular lowering motion in the treatment of disc dislocation without reduction and the dimensions before and after the treatment significantly varied statistically. Additionally, Delayme et al. [11], Ferrnandez-Ferro et al. [26], and Yang et al. [21] applied PRP with a beneficial effect on the functioning of the TMJs.

It is extremely important to be able to administer the drug to a place that needs healing and can be restored to function properly [10,11,15,17]. Self-application of the occlusal splints is much less effective and requires many months of occlusion. Daily activities such as speaking and taking food involves the movement of the jaw, and the pain associated with cases such as dislocation of the disc with a blockage makes these activities much more difficult. Therefore, the development of an algorithm to deal with the use of joint injections is so important in the therapeutic process of patients with complex TMD syndrome [19,26,28].

A certain limitation in the comparison of the obtained results of the tests carried out by several authors may be the different/variable parameters of the centrifugation of blood obtained in the ulnar vein. These differences concern the number of revolutions, centrifugation time, preparation of PRP, and the method of injection in the TMJ area. Studies of TMJ injections with PRP and HA have focused on decreased pain after injection in patients with both pain and limited mouth opening, secondary to displacement of the disc without reduction, and other clinical problems, such as arthritis and capsulitis [7,10,12,14,18,21].

## 5. Conclusions

The results of the comparative studies showed that the use of both substances positively affects selected clinical parameters in temporomandibular joints functional efficiency and decrease intensity of pain in temporomandibular joints is equally beneficial in the case of disc displacement without reduction. Both treatments show the same improvement, with no statistically significant difference between them.

## Figures and Tables

**Table 1:** Maximal interincisal distance obtained before and after management.

Before Management			After Management		
Maximal Interincisal Distance	Amount of Patients		Maximal Interincisal Distance	Amount of Patients	
	PRP Group	HA Group		PRP Group	HA Group
22 mm	12	10	40 mm	10	8
24 mm	14	8	42 mm	9	12
26 mm	8	10	43 mm	7	7
28 mm	5	5	44 mm	11	5
30 mm	7	9	45 mm	6	9
32 mm	4	8	46 mm	5	9

**Table 2:** Statistical parameters of the mean points of the temporomandibular joint’s pain before management.

Statistical Parameters	Mean Value	Median	Standard Deviation	Max-Min	*p*
PRP	6.80	7.0	1413	4–9	0.910
HA	6.76	7.0	1495	4–9	

**Table 3:** Statistical parameters of the mean points of temporomandibular joint’s pain after management.

Statistical Parameters	Mean Value	Median	Standard Deviation	Max-Min	*p*
PRP	2.74	3.00	0845	1–4	0.431
HA	2.88	3.00	0917	2–5	
